# Evolutionary breakpoint regions and chromosomal remodeling in *Harttia* (Siluriformes: Loricariidae) species diversification

**DOI:** 10.1590/1678-4685-GMB-2021-0170

**Published:** 2022-05-20

**Authors:** Geize Aparecida Deon, Larissa Glugoski, Terumi Hatanaka, Francisco de Menezes Cavalcante Sassi, Viviane Nogaroto, Luiz Antonio Carlos Bertollo, Thomas Liehr, Ahmed Al-Rikabi, Orlando Moreira, Marcelo de Bello Cioffi, Marcelo Ricardo Vicari

**Affiliations:** 1 Universidade Federal de São Carlos, Departamento de Genética e Evolução, São Carlos, SP, Brazil.; 2 Universidade Estadual de Ponta Grossa, Departamento de Biologia Estrutural, Molecular e Genética, Ponta Grossa, PR, Brazil.; 3 University Hospital Jena, Institute of Human Genetics, Jena, Germany.

**Keywords:** Chromosomal rearrangements, fish species, sex chromosome systems, whole chromosome painting

## Abstract

The Neotropical armored catfish genus *Harttia* presents a wide variation of chromosomal rearrangements among its representatives. Studies indicate that translocation and Robertsonian rearrangements have triggered the karyotype evolution in the genus, including differentiation of sex chromosome systems. However, few studies used powerful tools, such as comparative whole chromosome painting, to clarify this highly diversified scenario. Here, we isolated probes from the X_1_ (a 5S rDNA carrier) and the X_2_ (a 45S rDNA carrier) chromosomes of *Harttia punctata*, which displays an X_1_X_1_X_2_X_2_/X_1_X_2_Y multiple sex chromosome system. Those probes were applied in other *Harttia* species to evidence homeologous chromosome blocks. The resulting data reinforce that translocation events played a role in the origin of the X_1_X_2_Y sex chromosome system in *H. punctata*. The repositioning of homologous chromosomal blocks carrying rDNA sites among ten *Harttia* species has also been demonstrated. Anchored to phylogenetic data it was possible to evidence some events of the karyotype diversification of the studied species and to prove an independent origin for the two types of multiple sex chromosomes, XX/XY_1_Y_2_ and X_1_X_1_X_2_X_2_/X_1_X_2_Y, that occur in *Harttia* species. The results point to evolutionary breakpoint regions in the genomes within or adjacent to rDNA sites that were widely reused in *Harttia* chromosome remodeling.

## Introduction

Chromosome painting is a good tool for evolutionary investigation, once it may reveal how karyotypes have changed along their evolutionary history (Ried et al., 1998). Chromosome painting is based on fluorescence *in situ* hybridization (Ried *et al.*, 1998). Thus, the generation of probes from whole chromosomes or specific chromosomal regions obtained primarily by microdissection can be established (Guan et al., 1994, 1996). Chromosome painting can be used to identify homeologous segments and rearrangements during karyotype evolution (Yang et al., 2000; Ventura et al., 2009; Schemberger et al., 2011; Deng et al., 2013; Gokhman et al., 2019; Targueta et al., 2021). In teleosts, where banding patterns are not easily induced, a series of chromosomal rearrangements can be underestimated (Sharma et al., 2002). Accordingly, comparative chromosomal mapping can be a more appropriate method to reveal genomic rearrangements than the conventional cytogenetic bands in fishes (Nagamachi et al., 2010, 2013; Cioffi et al., 2011; Pucci et al., 2014; Oliveira et al., 2018).

Chromosome breakage in evolution can be a nonrandom event, and it has been observed that specific genomic regions have more propensity to break and trigger rearrangements than others (Pevzner and Tesler, 2003; Larkin et al., 2009). Genomic regions where the gene order has been conserved among species correspond to homologous synteny blocks (Murphy et al., 2005; Ruiz-Herrera et al., 2006). In this way, those small regions where the synteny has been disrupted by chromosomal reorganization may be named evolutionary breakpoint regions (Murphy *et al.*, 2005; Ruiz-Herrera *et al.*, 2006; Farré et al., 2011). The latter are enriched with repetitive sequences, including transposable elements, tandem repeats, and segmental duplications, providing conditions for non-allelic homologous recombination (Pevzner and Tesler, 2003; Bailey et al., 2004; Murphy *et al.*, 2005). It is suggested that these specific sites have been repeatedly used (i.e., reused) during chromosomal evolutionary processes (Ruiz-Herrera *et al.*, 2006; Carbone et al., 2009; Longo et al., 2009; Farré *et al.*, 2011). 

Loricariidae is one of the largest families of freshwater fishes, with over 1,000 valid species grouped in more than 100 genera and distributed throughout South and Central America (Reis et al., 2003; Fricke *et al.*, 2021). This family shows a substantial numerical and structural variation in karyotypes, mainly due to Robertsonian rearrangements (Artoni and Bertollo, 2001; Kavalco et al., 2004; Ziemniczak et al., 2012; Deon et al., 2020, 2022; Sassi et al., 2020), making it an outstanding group to investigate evolutionary processes (Mariotto et al., 2011; Barros et al., 2017; Glugoski et al., 2018, 2020). In some genera, the reuse of double-strand breaks suggests the occurrence of evolutionary breakpoint regions probable adjacent to rDNAs sites, as proposed for *Ancistrus* (Barros *et al.*, 2017), *Rineloricaria* (Glugoski *et al.*, 2018), and *Harttia* (Deon *et al.*, 2020).

*Harttia* includes a wide chromosomal variation in diploid numbers (2n = 52 - 62), karyotypes, number and position of the ribosomal clusters, and presence of sex chromosome systems (Blanco et al., 2017; Deon et al., 2020, 2022; Sassi et al., 2020, 2021). Until now, three different multiple sex chromosome systems have been reported in *Harttia*: i) an XX/XY_1_Y_2_ system in *H. carvalhoi*, *H. intermontana,* and *Harttia* sp. 1 (Blanco *et al.*, 2013; Deon *et al.*, 2020); ii) an X_1_X_1_X_2_X_2_/X_1_X_2_Y system in *H. duriventris, H. punctata* and *H. villasboas* (Blanco *et al.*, 2014; Sassi *et al.*, 2020) and iii) a neo XX/XY system in *H. rondoni* (Sassi *et al.*, 2020). Given the X_1_X_1_X_2_X_2_/X_1_X_2_Y sex chromosome system, *H. punctata* presents 2n=58 chromosomes in females and 2n=57 chromosomes in males, characterized by an exclusive submetacentric chromosome in the heterogametic sex (Blanco *et al.*, 2014). In this species, both ribosomal cistrons are related to sex chromosomes, with 5S rDNA sites found in the terminal region of the X_1_ pair in females and the X_1_ and Y chromosome in males, and with 45S rDNA sites being present in both X_2_ chromosomes in females and the single one in males (Blanco *et al.*, 2014). Chromosomal breaks and translocation events spanning the chromosomes 25 (X_1_) and 26 (X_2_) were proposed as ancestors of the Y chromosome (Blanco *et al.*, 2014). 

In this study, two probes for the whole X_1_ and X_2_ chromosomes of *H. punctata* (HPU-X_1_ and HPU-X_2_, respectively) were obtained by microdissection. The probes were used for comparative whole chromosome paintings (WCP) among 10 *Harttia* species to characterize homologous chromosome blocks and probable evolutionary breakpoint regions promoting karyotype differentiation. 

## Material and Methods

### Specimens and chromosome preparation

**Figure 1-:**
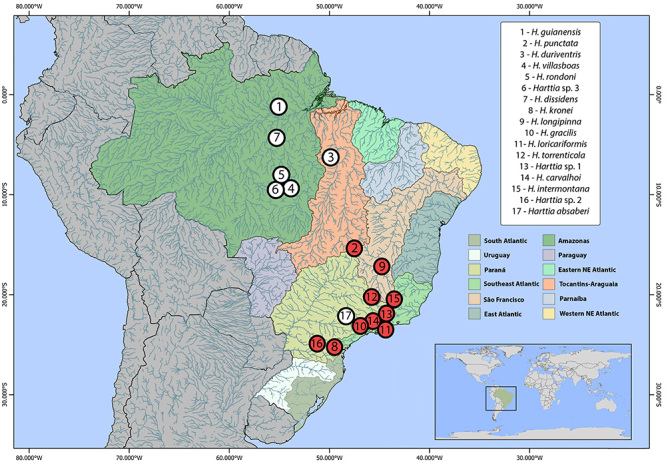
Partial map of South America highlighting the collection sites of *Harttia* species with cytogenetic data, which were numbered to their distribution into hydrographic basins according to clades proposed by phylogeny from Londoño-Burbano and Reis (2021): clade *i* - from the Guyana shield rivers - 1. *H. guianensis* (2n=58); clade *ii* - from the northern Brazilian rivers - 2. *H. punctata* (♀ 2n=58, X_1_X_1_X_2_X_2_/♂ 2n=57, X_1_X_2_Y)*,* 3. *H. duriventris* (♀ 2n=56, X_1_X_1_X_2_X_2_/♂ 2n=55, X_1_X_2_Y), 4. *H. villasboas* (♀ 2n=56, X_1_X_1_X_2_X_2_/♂ 2n=55, X_1_X_2_Y), 5. *H. rondoni* (2n=54, XX/XY), 6. *Harttia* sp. 3 (2n=54), 7. *H. dissidens* (2n=54); and clade *iii* - from the south/southeast Brazilian rivers - 8. *H. kronei* (2n=58)*,* 9. *H. longipinna* (2n=58)*,* 10. *H. gracilis* (2n=58), 11. *H. loricariformis* (2n=56), 12. *H. torrenticola* (2n=56)*,* 13. *Harttia* sp. 1 (♀ 2n=56, XX/♂ 2n=57, XY_1_Y_2_), 14. *H. carvalhoi* (♀ 2n=52, XX/♂ 2n=53, XY_1_Y_2_)*,* 15. *H. intermontana* (♀ 2n=52, XX/♂ 2n=53, XY_1_Y_2_)*,* 16. *Harttia* sp. 2 (2n=62), and 17. *H. absaberi* (2n=62). The collection sites of the species analyzed in this work are highlighted in red. Map created using QGis 3.4.3.

A total of 254 specimens of 10 *Harttia* species from South and Southeast Brazilian drainages here analyzed (Table 1, Figure 1). Fish were collected with the authorization of the Instituto Chico Mendes de Conservação da Biodiversidade (ICMBIO), System of Authorization and Information about Biodiversity (SISBIO-License Nos. 10538-3 and 15117-2), and National System of Genetic Resource Management and Associated Traditional Knowledge (SISGEN-A96FF09). All species, including two taxonomically undescribed species in the scientific literature, *Harttia* sp. 1 and *Harttia* sp. 2, were identified based on their morphological features by Dr. Oswaldo Oyakawa (curator of the fish collection of the Museu de Zoologia da Universidade de São Paulo - MZUSP). *Harttia* sp. 1 and *Harttia* sp. 2 karyotypes have already been published by Deon et al. (2020).

**Table 1-:** Collection sites of *Harttia* species, with their diploid number (2n) and sample sizes (N).

Species/ Sex chromosome system	2n	Sample collection in the map/ Locality	N
*H. punctata* (X_1_X_2_Y)	58♀/57♂	1. Bandeirinha river, Formosa - GO (15º19’25’’S 47º25’26’’W)	18♀,25♂
*H. longipinna*	58♀♂	2. São Francisco river, Pirapora - MG (17º21’22.8’’S 44º51’0.2’’W)	13♀,16♂
*H. torrenticola*	56♀♂	3. Araras stream, Piumhi - MG (20º16’15’’S 45º55’39’’W)	8♀, 6♂
*H. intermontana* (XY_1_Y_2_)	52♀/53♂	4. Piranga river, Carandaí - MG (20°59’34.0’’S 43°43’30.0’’W)	20♀, 13♂
*H. gracilis*	58♀♂	5. Machadinho stream, Santo Antônio do Pinhal - SP (22º48’31’’S 45º41’21’’W)	18♀,15♂
*Harttia* sp. 1 (XY_1_Y_2_)	56♀57♂	6. Macacos stream, Silveiras - SP (22°40’43.0”S 44°51’25.0”W)	10♀, 7♂
*H. loricariformis*	56♀♂	7. Paraitinga river, Cunha - SP (22º52’22’’S 44º51’0.2’’W)	7♀, 3♂
*H. carvalhoi* (XY_1_Y_2_)	52♀/53♂	8. Grande stream, Pindamonhangaba - SP (22º47’8’’S 45º27’19’’W)	17♀, 12♂
*H. kronei*	58♀♂	9. Açungui river, Campo Largo - PR (25º22’44’’S 49º39’0.8’’W)	10♀, 5♂
*Harttia* sp. 2	62♀♂	10. Barra Grande river, Prudentópolis - PR (24°58’40.72’’S 51°7’34.25’’W)	17♀, 11♂

SP = São Paulo; MG = Minas Gerais; PR = Paraná; GO = Goiás Brazilian States.

Mitotic chromosomes were obtained from kidney cells, according to Bertollo et al. (2015). The experiments were conducted under the Ethics Committee on Animal Experimentation of the Universidade Federal de São Carlos approval (Process number CEUA 1853260315). Cell preparations were dropped onto clean glass slides at 55 °C and stained with Giemsa solution 5%. 

### Chromosome microdissection, probes, and labeling

Fifteen copies of the X_1_ and X_2_ chromosomes of *H. punctata* were isolated by microdissection and amplified using the procedure described in Yang et al. (2009). Their obtained probes HPU-X_1_ and HPU-X_2_ were then labeled with Spectrum Orange-dUTP and Spectrum Green-dUTP (Vysis, Downers Grove, USA), respectively, in a secondary DOP-PCR, using 1 μl of the primarily amplified product as a template DNA, following Yang and Graphodatsky (2009). All the microdissection procedures were performed in the Molecular Cytogenetics Laboratory at the Institut für Humangenetik at Universitätsklinikum Jena, Germany.

### Fluorescence *in situ* hybridization (FISH) for WCP

Two female and two male mitotic preparations for each species were submitted to WCPs. Slides were prepared and pre-treated according to Yang et al. (2009) and denatured in 70 % formamide/2xSSC for 3 min at 72 °C. For each slide, 12 μl of hybridization solution (containing 0.2 μg of each labeled probe, 50 % formamide, 2xSSC, and 10 % dextran sulfate) were denatured for 10 minutes at 75 ^o^C and allowed to pre-hybridize for 1h at 37 ^o^C. To block the hybridization of high-copy repeat sequences, 20 μg of C_0_t-1 DNA, directly isolated from *H. punctata* male genome, were prepared according to Zwick et al. (1997). Hybridization was done for 48 h at 37 °C in a moist chamber. Post-hybridization washes were performed in 1xSSC for 5 min at 65 °C and 5min in 4xSSC/Tween at room temperature. Chromosomes were counterstained with 4’, 6-diamidino-2-phenylindole (DAPI) in Vectashield mounting medium (Vector, Burlingame, CA, USA). 

### FISH for 5S and 18S rDNA

Two tandemly arrayed rDNA probes were obtained by PCR from the nuclear DNA of *Harttia intermontana*. The 5S rDNA probe included 120 base pairs (bp) of the 5S rRNA transcript region and 200 bp of a non-transcribed spacer, isolated according to Martins and Galetti (1999) using the primers A (5’-TCAACCAACCACAAAGACATTGGCAC-3’) and B (5’-TAGACTTCTGGGTGGCCAAAGGAATCA-3’). The 18S rDNA probe contained a 1,400 bp segment of the 18S rRNA gene and was isolated following Cioffi et al. (2009) using the primers 18SF (5’-CCGAGGACCTCACTAAACCA-3’) and 18SR (5’-CCGCTTTGGTGACTCTTGAT-3’). Both probes were directly labeled with the Nick-Translation mix kit (Jena Bioscience, Jena, Germany): the 5S rDNA with ATTO550-dUTP (Jena Bioscience) and the 18S rDNA with AF488-dUTP (Jena Bioscience), following the manufacturer’s manual. FISH experiments followed the methodology described in Yano et al. (2017). 

### Images capture and processing

Metaphase plates were captured using an Olympus BX50 light microscope (Olympus Corporation, Ishikawa, Japan) with a CoolSNAP camera. The images were processed using the Image-Pro Plus 4.1 software (Media Cybernetics, Silver Spring, MD, USA). Twenty to thirty metaphases were analyzed per sampled individual for WCP and FISH signals detection.

## Results

**Figure 2 -:**
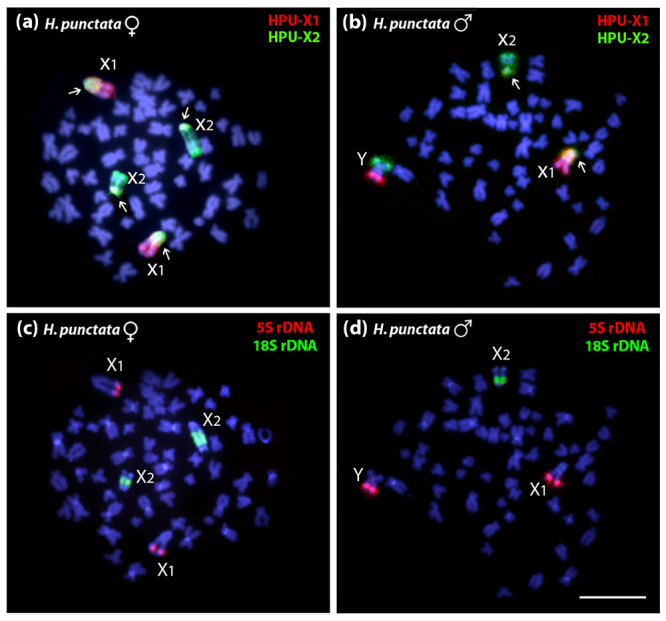
Fluorescence *in situ* hybridization results using the HPU-X_1_ (red) and HPU-X_2_ (green) probes in female (2n=58) and male (2n=57) chromosomes of *H. punctata*, and sequential FISH with 5S rDNA (red) and 18S rDNA (green) probes. The white arrows indicate overlapping signals and represent DNA segments in common. Chromosomes were counterstained with DAPI (blue). Bar = 5 µm.

HPU-X_1_ and HPU-X_2_ probes hybridized to *H. punctata* X_1_ and X_2_ chromosomes, and results revealed that a DNA segment in common was present in their proximal regions (Figure 2a). In male karyotype, HPU-X_1_ and HPU-X_2_ probes detected the monovalent X_1_ and X_2,_ and also the Y chromosome was stained by HPU-X_1_ probe in its distal region of the long arm (q) and by HPU-X_2_ signal in the short arm (p) (Figure 2b). A sequential FISH using the 5S and 18S rDNA probes efficiently detected 5S rDNA on X_1_ and Y chromosomes and 18S rDNA on X_2_ chromosome (Figure 2c, d). 

**Figure 3 -:**
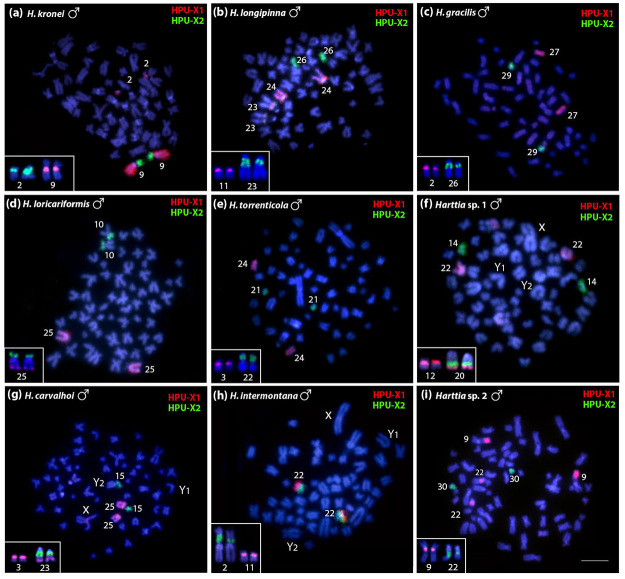
Male metaphases of *Harttia* after WCP using the HPU-X_1_ (red) and HPU-X_2_ (green) probes from *Harttia punctata* for comparative analyses: (a) *H. kronei* (2n=58), (b) *H. longipinna* (2n=58), (c) *H. gracilis* (2n=58), (d) *H. loricariformis* (2n=56), (e) *H. torrenticola* (2n=56), (f) *Harttia* sp. 1 (2n=57, XY_1_Y_2_), (g) *H. carvalhoi* (2n=53, XY_1_Y_2_), (h) *H. intermontana* (2n=53, XY_1_Y_2_), and (i) *Harttia* sp. 2 (2n=62). The chromosomes displaying the 5S rDNA (red) and 18S rDNA (green) sites are highlighted in the boxes. Chromosomes were counterstained with DAPI (blue). Bar = 5 µm.

**Figure 4 -:**
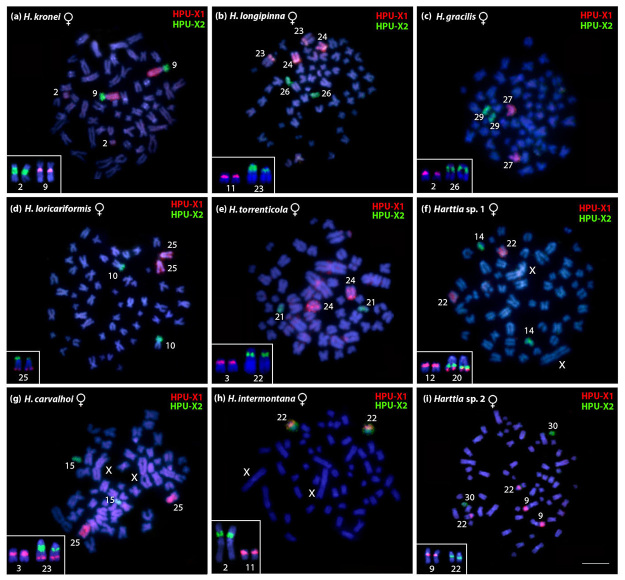
Female metaphases of *Harttia* after WCP using the HPU-X1 (red) and HPU-X2 (green) probes from *Harttia punctata* for comparative analyses: (a) *H. kronei* (2n=58), (b) *H. longipinna* (2n=58), (c) *H. gracilis* (2n=58), (d) *H. loricariformis* (2n=56), (e) *H. torrenticola* (2n=56), (f) *Harttia* sp. 1 (2n=56, XX), (g) *H. carvalhoi* (2n=52, XX), (h) *H. intermontana* (2n=52, XX), and (i) *Harttia* sp. 2 (2n=62). The chromosomes displaying the 5S rDNA (red) and 18S rDNA (green) sites are highlighted in the boxes. Chromosomes were counterstained with DAPI (blue). Bar = 5 µm.

**Figure 5-:**
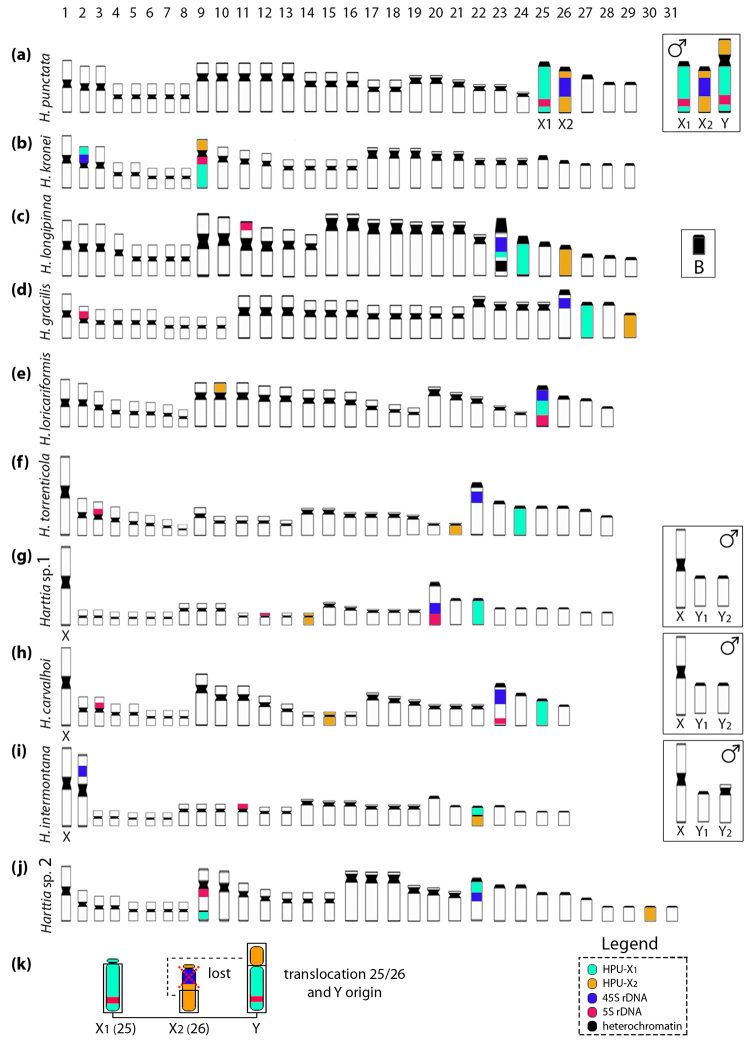
Idiograms representative of the *Harttia* species analyzed in this study with HPU-X_1_, HPU-X_2_, 5S rDNA, and 18S rDNA probes. In (a) *H. punctata* idiogram demonstrating the structure of the X_1_ and X_2_ chromosome probes used for comparative whole chromosome paintings in this study (5S rDNA site on X_1_ and 45S rDNA on X_2_); (b-j) idiograms of the nine *Harttia* species (*H. kronei*, *H. longipinna*, *H. gracilis*, *H. loricariformis*, *H. torrenticola*, *Harttia* sp. 1, *H. carvalhoi*, *H. intermontana*, and *Harttia* sp. 2, respectively) from South and Southeast of Brazil demonstrating the HPU-X_1_ and HPU-X_2_ homeologs blocks; and (k) schematic representation based on Blanco et al. (2014) of the rearrangements between the 25 and 26 male chromosomes giving rise to the X_1_X_1_X_2_X_2_/X_1_X_2_Y sex chromosome system of *Harttia punctata*, as clarified by chromosomal painting.

Cross-species FISH using the two WCPs was performed among all the nine other species from Table 1 (Figures 3 and 4), and their signals were compared to *H. punctata* karyotype (Figure 5a). In *H. kronei,* the HPU-X_1_ painted chromosome 9q and distal region of chromosome 2p, while HPU-X_2_ painted chromosome 9p (Figures 3a, 4a, and 5b). The 5S and 18S rDNAs were mapped to the proximal regions of chromosomes 9q and 2p, respectively (Figures 3a, 4a, and 5b). In *H. longipinna,* the HPU-X_1_ probe hybridized to chromosome 24q and the adjacent regions to the secondary constriction of chromosome 23, while the HPU-X_2_ probe hybridized to chromosome 26 (Figures 3b, 4b, and 5c). Besides that, the 5S and 18S rDNAs were detected in proximal regions of chromosomes 11p and 23q, respectively (Figures 3b, 4b, and 5c). In *H. gracilis*, HPU-X_1_ and HPU-X_2_ hybridized to pairs 27 and 29, respectively (Figures 3c, 4c, and 5d). The 5S and 18S rDNAs sites were in situ localized to the proximal regions of chromosomes 2p and 26q, respectively (Figures 3c, 4c, and 5d). 

*Harttia loricariformis* showed the HPU-X_1_ probe hybridized to chromosome 25q, the HPU-X_2_ in chromosome 10p, the 5S rDNA in the distal region of 25q, and the 18S rDNA probe located in the distal region of 25p (Figures 3d, 4d, and 5e). *Harttia torrenticola* showed the HPU-X_1_ hybridized to chromosome 24, the HPU-X_2_ probe to chromosome 21, and the 5S and 18S rDNAs in the proximal regions of chromosomes 3p and 22q, respectively (Figures 3e, 4e, and 5f). In *Harttia* sp. 1, the HPU-X_1_ probe hybridized to chromosome 22 and the HPU-X_2_ probe to chromosome 14 (Figures 3f, 4f, and 5g). The 5S rDNA was detected in the proximal region of chromosome 12p and the distal region of chromosome 20q, the last chromosome also bearing the 18S rDNA cluster (Figures 3f, 4f, and 5g). 

*Harttia carvalhoi* showed the HPU-X_1_ probe hybridized to chromosome 25 and the HPU-X_2_ probe to chromosome 15 (Figures 3g, 4g, and 5h). The 5S rDNA probe hybridized to the proximal region of chromosome 3p and the distal region of chromosome 23q, while the 18S rDNA probe hybridized to the proximal region of 23q (Figures 3g, 4g, and 5h). In *H. intermontana,* the HPU-X_1_ and the HPU-X_2_ probes hybridized to the same chromosome, i.e., 22p and 22q regions, respectively (Figures 3h, 4h, and 5i). The 5S and 18S rDNA probes hybridized to the proximal regions of the chromosomes 11p and 2p, respectively (Figures 3h, 4h, and 5i). *Harttia* sp. 2 showed the HPU-X_1_ probe hybridized to the distal middle region of chromosome 9q and to the proximal region of the 22q, while the HPU-X_2_ probe hybridized to chromosome 30 (Figures 3i, 4i, and 5j). The 5S and 18S rDNAs were evidenced in the proximal regions of chromosomes 9q and 22q, respectively (Figures 3i, 4i, and 5j). All the results obtained with the HPU-X_1_, HPU-X_2_, 5S rDNA and 18S rDNA probes location were summarized in Table 2.

**Table 2 -:** Localization of WCP and rDNA probes analyzed in *Harttia* species.

Species	HPU-X_1_ probe	HPU-X_2_ probe	5S rDNA probe	18S rDNA probe
*H. punctata ***♂****	Chr. 25 (X_1_) and Y	Chr. 26 (X_2_) and Y	25q distal	26q proximal
*H. punctata ♀*	Chr.25 (X_1_)	Chr. 26 (X_2_)	25q distal	26q proximal
*H. longipinna ♀ ***♂****	24q and 23q proximal	Chr. 26	11p proximal	23q proximal
*H. torrenticola ♀ ***♂****	Chr. 24	Chr. 21	3p proximal	22q proximal
*H. intermontana ♀ ***♂****	22p	22q	11p proximal	2p proximal
*H. gracilis ♀ ***♂****	Chr. 27	Chr. 29	2p proximal	26q proximal
*Harttia sp. 1 ♀ ***♂****	Chr. 22	Chr. 14	12p proximal and 20q distal	20q proximal
*H. loricariformis ♀ ***♂****	25q	10p	25q distal	25p distal
*H. carvalhoi ♀ ***♂****	Chr. 25	Chr. 15	3p proximal and 23q distal	23q proximal
*H. kronei ♀ ***♂****	9q and 2p distal	9p	9q proximal	2p proximal
*Harttia sp. 2 ♀ ***♂****	9q distal and 22q proximal	Chr. 30	9q proximal	22q proximal

p = short arms; q = long arms; Chr. = chromosome.

## Discussion

A combined molecular and morphological phylogeny of the Harttiini and Farlowellini tribes recognized three distinct clades for the *Harttia* genus (Londoño-Burbano and Reis, 2021). These clades grouped species according to their South American distribution: (i) from the Guyana shield rivers; (ii) from the northern Brazilian rivers; and (iii) from the Brazilian south/southeast rivers (Londoño-Burbano and Reis, 2021). Karyotype evolution scenarios have been proposed in *Harttia*, anchoring the chromosomal data to the Harttiini phylogeny (Blanco et al., 2017; Deon et al., 2020; Sassi et al., 2020, 2021). In all scenarios, extensive events of chromosomal remodeling have been identified in *Harttia*, changing the 2n, chromosome morphologies and triggering sex chromosome systems origin independently in each clade (Blanco *et al.*, 2017; Deon *et al.*, 2020, 2022; Sassi *et al.*, 2020, 2021), as also identified in this study. 

**Figure 6-:**
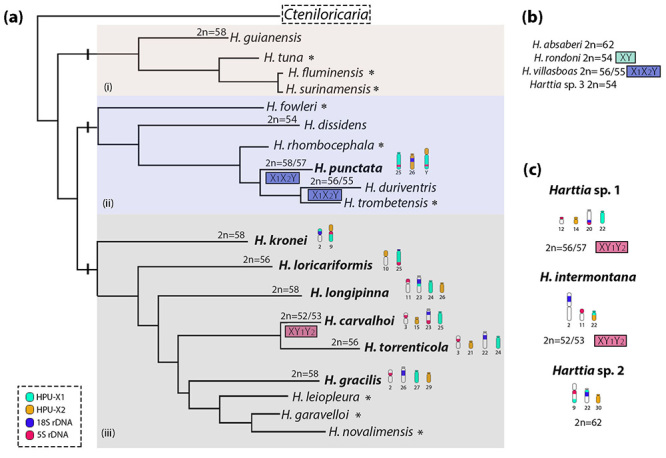
Schematic representation of the phylogenetic relationships among *Harttia* species from Londoño-Burbano and Reis (2021) integrated with cytogenetic data. In (a), phylogenetic relationships with the representation of the *Harttia* clades i (Guyana shield rivers)*,* ii (northern Brazilian rivers), and iii (south/southeast Brazilian rivers). On the branches side, idiogramatic representation of the chromosomes bearing 5S rDNA, 45 rDNA, HPU-X_1_ and HPU-X_2_ homeologous blocks. These regions triggered extensive chromosomal remodeling in the *Harttia* lineage. In (b), *Harttia* species with cytogenetic data but not present on original phylogeny. In (c) an idiogramatic representation of the chromosomes bearing 5S rDNA, 45 rDNA, HPU-X_1_ and HPU-X_2_ homeologous blocks in species not present on original phylogeny (*Harttia* sp.1, *H. intermontana*, and *Harttia* sp. 2), but that had data analyzed in this study. * Species without cytogenetic characterization.

Both *H. punctata* derived probes (HPU-X_1_ and HPU-X_2_) were able to detect homeologous chromosome blocks in *Harttia* species, highlighting chromosomal rearrangements that occurred during lineage evolution. WCP has also been used for genomic comparisons to detect homeologous blocks among different species (Ventura et al., 2009). Regarding the X_1_X_1_X_2_X_2_/X_1_X_2_Y sex chromosome system origin in *H. punctata*, the HPU-X1 and XPU-X2 hybridizations corroborate the proposal of Blanco et al. (2014). In this proposal, one translocation event involving chromosomes 25 and 26 (now representing chromosomes X_1_ and X_2_, respectively), with proximal segments lost, gave rise to the Y chromosome (Blanco *et al.*, 2014, Figure 5k). It is also relevant to point out that no positive signs of the HPU-X_1_ and HPU-X_2_ probes were found on the XY_1_Y_2_ chromosomes of *H. carvalhoi, Harttia* sp.1, and *H. intermontana* from the Brazilian south/southeast clade (Figures 5 and 6). This data indicates an independent origin for the two models of the multiple sex chromosome systems - X_1_X_2_Y and XY_1_Y_2_ - that occur in the *Harttia* genus, an evolutionary route also proposed for some other teleost groups (Devlin and Nagahama, 2002; Cioffi et al., 2013; Sember et al., 2018).

An ancestral karyotype with 2n=58 chromosomes and without a differentiated sex chromosome system is proposed to the *Harttia* lineage (Blanco et al., 2017). Based on phylogenetic data (Covain et al., 2016; Londoño-Burbano and Reis, 2021) and the description of the 2n=58 chromosomes in the sister group *Farlowella* (Marajó et al., 2018), the data reinforces the proposal of a putative ancestral karyotype with 2n=58 chromosomes for the *Harttia* clade iii (Figure 6). *Harttia punctata* belongs to the clade (ii), and their X_1_ and X_2_ chromosomes were applied in WCP in species from the clade (iii) of *Harttia* to evaluate the chromosomal diversification. Following a probable diversification scenario in species from the clade (iii), *H. kronei* presented *H. punctata* X_1_ in the distal regions of chromosome 2p and 9q, while the arm 9p represents chromosome X_2_ (Figures 5 and 6). Besides that, the proximal regions of the chromosomes 2p and 9q are arranged by 45S and 5S rDNAs, respectively (Figures 5 and 6). The WCPs and rDNA *in situ* localization suggest sites prone to break within or adjacent to the rDNA sites were widely reused throughout the chromosomal evolution of *Harttia*, as can be observed in species from the clade iii. 

Chromosomal breaks in the centromere region of chromosomes 2 and 9 from *H. kronei* followed by rearrangements could originate the chromosomes 10 and 25 in *H. loricariformis*. Since double-strand breaks close to rDNA sites have occurred, the chromosome arm 10p from *H. loricariformis* keeps a homeologous block with 9p of the *H. kronei* (Figures 5 and 6). At the same time, a fusion of the chromosome arms 2p and 9q from *H. kronei* could organize the acrocentric pair 25 bearing 5S and 45S rDNA sites of the *H. loricariformis* (Figures 5 and 6). In this pathway, the chromosomes 10 and 25 are not evolved in the 2n reduction to 56 chromosomes in *H. loricariformis*. A Robertsonian fusion could explain the 2n decrease in this species once an interstitial telomeric site was proposed in a large subtelocentric pair (Blanco et al., 2017). 

In *H. longipinna* lineage, chromosomal breaks close to rDNA sites rearranged 5S rDNA and 45S rDNA clusters to chromosomes 11 and 23, respectively (Figures 5 and 6). In addition, chromosome fission could originate acrocentrics 24 and 26 carrying the HPU-X_1_ and HPU-X_2_ homeologous blocks, respectively (Figures 5 and 6). Thus, the 2n=58 chromosomes in *H. longipinna* and *H. gracilis* could be an evolutionary recurrence feature. It is interesting to note, although additional chromosomal changes occurred in chromosomes possessing 5S rDNA, 45 rDNA, HPU-X_1_ and HPU-X_2_ homeologous blocks, these four chromosomes were kept in *H. longipinna*, *H. gracilis*, *H. torrenticola*, *Harttia* sp.1, and *H. carvalhoi* (Figures 5 and 6). Besides that, the 2n=56 of *H. torrenticola* had an independent mechanism once a Robertsonian fusion was proposed in the origin of its pair 1 (Blanco et al., 2017).

*Farlowella* species (a sister group of *Harttia*) have single 45S rDNA and 5S rDNA sites (Marajó et al., 2018). Based on this description, the *Harttia* sp. 1 and *H. carvalhoi* karyotypes presented an extra 5S rDNA site that could have emerged by gene units gain and rearrangements. In these species, a transposition could rearrange the extra site to the syntenic condition with 45S rDNA (Figures 5 and 6). In addition, comparing *H. carvalhoi* and *Harttia* sp. 1 karyotypes it is possible to detect an inversion relocating the syntenic 5S and 18S rDNA sites (Figures 5 and 6). *Harttia intermontana* lineage showed probable translocations to originate the metacentric 2 bearing the 45S rDNA site and the chromosome 22 bearing the HPU-X_1_ and HPU-X_2_ homeologous blocks (Figures 5 and 6). Yet, transpositions or translocations rearranged rDNA sites and HPU-X_1_ and HPU-X_2_ homeologous blocks in the *Harttia* sp. 2 karyotype (Figures 5 and 6). All data demonstrating extensive chromosomal remodeling involving double-strand breaks and rearrangements reinforce the proposal of evolutionary breakpoint regions close to rDNA sites in *Harttia* lineage (Deon et al., 2020). 

Ribosomal clusters as promoters of chromosomal reorganization, mainly those located in the pericentromeric regions, have been the focus of previous studies on Robertsonian rearrangements (Sullivan et al., 1996; Rosa et al., 2012; Barros et al., 2017; Glugoski et al., 2018). The rDNA sites have been associated with critical chromosomal breakpoints given some features, as follow: tandem arrangements, usually pericentromeric or subterminal locations; ability to transpose; high rates of intra- and inter-chromosomal recombination (Cazaux et al., 2011), in addition to intense gene expression activity (Huang et al., 2008). Several types of rearrangements may result from chromosomal breaks, leading to rapid changes in the distribution of the rDNA sites among closely related species (Datson and Murray, 2006; Degrandi *et al.*, 2014). Our WCP data in *Harttia* species also indicate that adjacent regions to the rDNAs sites have been extensively reused in the chromosomal diversification of this genus. 

The association between chromosomal breaks and rDNA sites is well documented in rodents, especially in *Mus* species (Cazaux et al., 2011). In fish, although highly diverse karyotypes occur among its representatives, few studies portray chromosomal remodeling and its causes. Some of them, using *in situ* hybridization with rDNA probes, indicated that the distribution and dispersion of these sequences may have contributed to genomic diversification among Loricariidae species (Kavalco et al., 2004; Rosa et al., 2012; Errero-Porto et al., 2014; Barros et al., 2017; Primo et al., 2017; Glugoski et al., 2018, 2020). In *Harttia*, the present data evidence evolutionary breakpoint regions inside or adjacent to the 5S and 18S rDNA sites and their reuse triggering several chromosomal rearrangements during the evolutionary story of this lineage. 

The current results cannot explain several chromosomal rearrangements that had occurred during the karyotype evolution of *Harttia*. Among them, the diversified diploid number in *Harttia* sp. 2, the origin of the largest metacentric pair in *H. carvalhoi, H. intermontana, H. torrenticola* and *Harttia* sp.1, and the differentiation of the XY_1_Y_2_ sex chromosome system in species from the Brazilian south/southeast region. However, our data were able to clarify the reuse of evolutionary breakpoint regions inside or to surround rDNA sites in promoting several rearrangements of homeologous chromosome blocks, and so triggering an extensive chromosomal remodeling among *Harttia* species.
